# Study on Bonding Characteristics of Polymer Grouted Concrete-Soil Interface

**DOI:** 10.3390/polym16152207

**Published:** 2024-08-02

**Authors:** Lina Wang, Xiaodong Yang, Yueliang Diao, Chengchao Guo

**Affiliations:** 1 School of Water Conservancy and Transportation, Zhengzhou University, Zhengzhou 450001, China; 2 Hefei Commumications Investment Hetong Expressway Co., Ltd., Hefei 231200, China; 3 School of Civil Engineering, Sun Yat-sen University, Guangzhou 510275, China

**Keywords:** diaphragm wall, permeable polymer, interfacial grouting, direct shear test, microstructure

## Abstract

The issue of interfacial shear damage has been a significant challenge in the field of geotechnical engineering, particularly in the context of diaphragm walls and surrounding soils. Polymer grouting is a more commonly used repair and reinforcement method but its application to interface repair and reinforcement in the field of geotechnical engineering is still relatively rare. Consequently, this paper presents a new polymer grouting material for use in grouting reinforcement at the interface between concrete and soils. The bonding characteristics and shear damage mode of the interface after grouting were investigated by the direct shear test, and the whole process of interface shear damage was investigated by digital image correlation (DIC) technology. Finally, the reinforcement mechanism was analyzed by microscopic analysis. The results demonstrate that the permeable polymer is capable of effectively filling the pores of soil particles and penetrating into the concrete-soil interface. Through a chemical reaction with water in the soil, the polymer cements the soil particles together, forming chemical adhesion at the interface and thereby achieving the desired reinforcement and repair effect. In the shear process, as the normal stress increased, the horizontal displacement and horizontal compressive strain at the distal end of the loading end decreased, while the maximum vertical displacement and maximum vertical strain of the cured soil also decreased. The results of scanning electron microscopy (SEM) demonstrated that the four groups of test polymers exhibited a reduction in soil porosity of 53.47%, 58.79%, 52.71%, and 54.12%, respectively. Additionally, the form of concrete-soil interfacial bonding was observed in the concrete-cohesive layer-cured soil mode. The findings of this study provide a foundation for further research on diaphragm wall repair and reinforcement.

## 1. Introduction

Due to the different material properties between the concrete structure and the surrounding soil, the interface is most prone to defect problems, so the study of concrete-soil interface reinforcement is of great significance in practical engineering [1,2,3]. Grouting repair technology is a more widely used repair technology, which is increasingly widely used in the field of construction and geotechnology, and polymer grouting repair technology is one of the important aspects; polymer grouting repair for concrete-soil-rock interface damage repair and reinforcement provides a novel approach to the issue at hand [4,5,6,7].

In academic research, grouting is a method for reinforcing and repairing the soil-rock interface without excavation, providing a convenient and eco-friendly approach to improve the mechanical characteristics [8,9,10,11]. There are two main categories of grouting materials: cement slurries and chemical slurries. The widespread use of cement slurry is attributed to its abundant availability, affordability, lack of toxicity, and straightforward construction procedure. By modifying the water-cement ratios, one can attain a considerable increase in compressive strength. However, its shortcomings such as long setting time, easy segregation of water secretion shrinkage and cracking, and large stiffness limit its further application [12,13,14,15]. Polyurethane is a polymer composed of polyisocyanate and polyether polyol or polyester polyol along with other raw materials [16,17]. The main advantages of polyurethane grouting materials are low viscosity, short reaction time, good impermeability, and durability. These advantages make them extensively utilized for strengthening and restoring high-speed railways, soil dams, subgrades, pavements, underground pipelines, and various other projects [18,19,20]. However, the penetration depth to the soil is limited due to the rapid reaction expansion characteristics of expansive polymers [21]. Based on this, a permeable polymer grouting material was developed, which is environmentally friendly and stable. The use of penetration grouting technology enables the slurry to seep into the soil pores under pressure without disturbing the strata. This process helps remove air and water from the soil while also cementing the soil particles together. The ultimate goal is to achieve the effect of strengthening the soil. Consequently, it is necessary to explore the mechanical properties of the soil-rock interface reinforced by permeable polymer.

Increasing the bonding performance of the interface directly affects the structural durability and reliability, and increasing the strength of the interface by grouting is one of the important means. Many scholars have conducted research on interface grouting. Hossain et al. [22] investigated the mechanical properties of the contact surface between cement slurry and granite by means of direct shear tests. Hongyuan Fang et al. [23] carried out direct shear tests on the interface between polymer and concrete based on polymer grouting for repairing underground pipe dehiscence. Lei Qin et al. [24] carried out shear tests on polymer-concrete composite specimens with different surface roughness and polymer density by means of cyclic shear tests. Papastergiou et al. [25] investigated the structural properties of the restrained grouting interface of a new type of steel-concrete joint through cyclic shear tests. Zhang et al. [26] conducted an investigation into the shear characteristics of the grouted interface between the soil and the steel plate and subsequently compared the results with those obtained from shear tests on in situ plastic clay and gravelly clayey soils. Zhang et al. [27] constructed a microscopic binding interface model of modified bentonite based on the molecular dynamics simulation method and investigated the interfacial shear mechanical properties under different working conditions. Combined with the above analysis, the interfacial mechanical properties between different materials after grouting have been widely studied, but the interfacial bonding performance of the diaphragm wall concrete structure and the surrounding soil after grouting is affected by many factors, and with the increase in ultra-deep diaphragm wall construction projects, the traditional grouting method is difficult to effectively grout and reinforce the diaphragm wall projects with a large burial depth and complex hydrogeological conditions. The infiltration grouting technology can be used to grout the soil and different material interfaces without disturbing the stratum structure, and the infiltration grouting technology adopts the permeable grouting material that can penetrate into the soil where the pore structure is even smaller, which can effectively improve the grouting reinforcement effect, but combined with the existing research, the research of the bonding characteristics of the concrete structure and soil interfaces of the underground diaphragm wall based on infiltration grouting is relatively incomplete, and it is not clear how infiltration grouting can be applied to the concrete and soil interfaces. It is not clear what the reinforcement effect and the intrinsic mechanism of infiltration grouting on the interface between concrete and soil are.

In order to more fully study the bonding characteristics of the concrete-soil interface after penetration grouting, through the indoor interface grouting method, and through the penetrating polymer soil-rock interface direct shear performance test, combined with DIC (Digital Image Correlation) measurement technology, the whole process of the interface damage was analyzed to investigate the evolution of interface shear damage, and at the same time, through the ImageJ analysis of the interface before and after grouting, and finally, based on SEM, to carry out microtests to study the reinforcement mechanism.

## 2. Materials and Methods

### 2.1. Test Soil

The soil for this test is taken from China; the soil is taken by manual excavation, and the depth of the soil is deep after 4.5 m. According to the relevant test methods in the test standard, the basic physical property indexes of the soil can be obtained as shown in Table 1.

**Figure 1:**
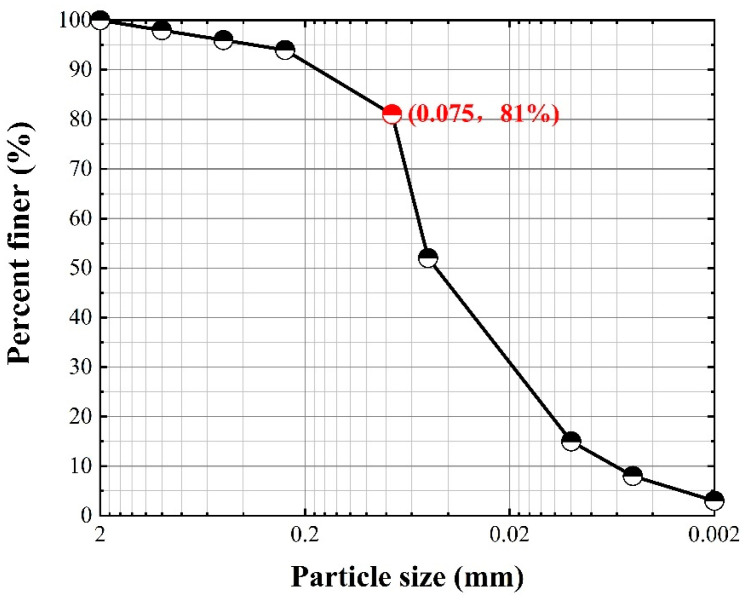
Grain size distribution of soils.

The test soil for particle size analysis, in which the particle size is greater than 0.075 mm above the soil particles, uses the vibration sieving method. For the vibratory sieving method, 500 g of dried soil samples for sieving must first be weighed, and then we calculated the corresponding percentage. According to the results of the particle size analysis of the test soil, the particle size in the range of 0.002 mm–0.075 mm particle mass is 81% of the total mass; the soil used in this test is powdered soil, and the particle grading curve of the test soil is shown in Figure 1.

### 2.2. Polymer Grouting Materials

**Figure 2:**
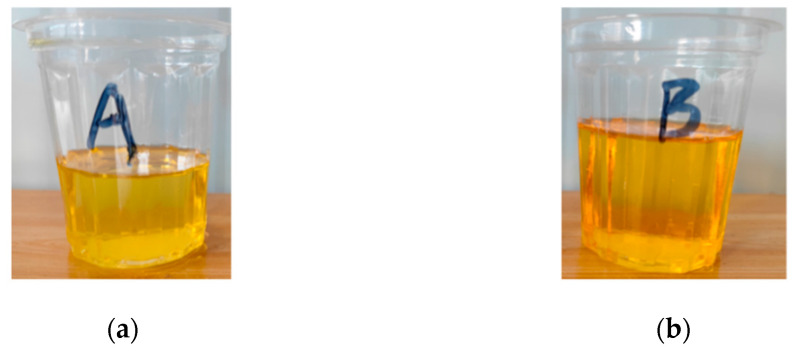
Permeable polymer materials: (**a**) component A; and (**b**) component B.

The grouting material used in this test is a permeable polymer that can react with water, which was developed by the team independently, and was made by homogeneous mixing of component A and component B according to the mass ratio of 1:1 [28,29,30]. The polymer component A is composed primarily of polyether polyol, surfactant, and dimethylformamide (nHO~OH), while component B is primarily composed of polymethylene polyphenyl polyisocyanate and toluene diisocyanate. Both component A and component B are yellow liquids. Figure 2 shows the actual photos of component A and component B.

The permeable polymer grouting material used in this paper has low viscosity, good permeability and mobility, and high cementation strength and durability, which can effectively fill the pore space of the particles in the soil. The permeable polymer grouting material mainly reacts with the water in the soil to generate cementing material and enhances the physical properties of the injected medium through its own strength and cementing effect. Before grouting, component A and component B are poured into the storage barrel of the grouting equipment according to the mass ratio of 1:1, stirred evenly in the storage barrel for a period of time to ensure that the two-component solution is fully mixed, and then injected into the mold. The reaction time of the material after grouting is about 10 min, and the curing time is about 30 min. Table 2 shows the basic performance parameters of the grouting material.

**Figure 3:**
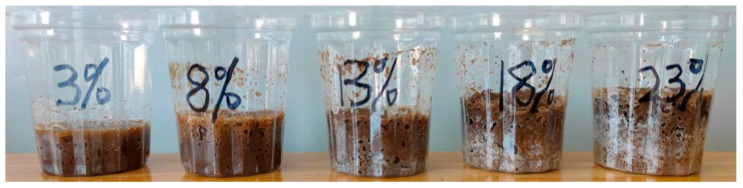
Product of mixing equal masses of polymers and soils with different water contents.

Figure 3 shows the products of the reaction of this permeable polymer grouting material mixed with different water content test soil in an equal mass ratio. A small amount of gas is generated after the reaction between the permeable polymer and the water in the soil, and the volume of the specimen is slightly swollen after the reaction, and the volume expansion rate increases with the increase in water content; the internal porosity of the cured soil increases, and the color of the cemented material changes from dark to white, and at the same time, the viscoelasticity of the specimen after the reaction is increased, and the compressibility is increased.

### 2.3. Concrete Materials

**Figure 4:**
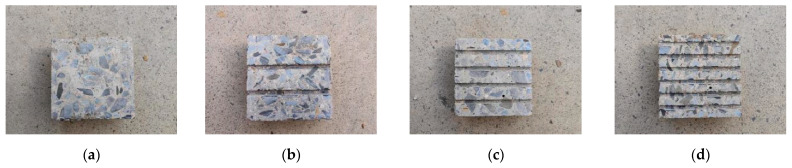
Interface roughness treatment:(**a**) smoothness; (**b**) roughness 1; (**c**) roughness 1; (**d**) roughness 4.

Figure 4 shows the interface roughness treatment. The designed strength grade of the concrete used in this test is C20, C30, C40, and C50, and the casting specification is 100 mm × 100 mm × 100 mm, which is poured manually. The unmolded concrete specimen was put into the standard curing room to be cured for 28 days, and after the curing was completed, the concrete block of casting specification was cut into the test specification of 100 mm × 100 mm × 50 mm by using the numerical control machine. The roughness of the limestone surface can be expressed by the surface roughness index *JCR.* The surface roughness index *JCR* proposed in the paper of Barton and Choubey [31] is used to quantify the roughness of the concrete surface, and the calculation of *JCR* in the paper of Du et al. [32,33] is shown in Equation (1). Based on Equation (1), the *JCR* value of the interface roughness can be calculated. Table 3 shows the interface roughness table.

(1) $$JCR=49.2114e^{\frac{29L_{0}}{450L_{n}}}arctan\left(\frac{8R_{y}}{L_{0}}\right)$$

where *L*_0_ is the horizontal length of the nodal profile in mm; *L_n_* is the actual length of the nth nodal profile in mm; *R_y_* is the amplitude of the profile with a sampling length of *L_n_* in mm.

### 2.4. Grouting Test and Specimen Preparation Methods

**Figure 5:**
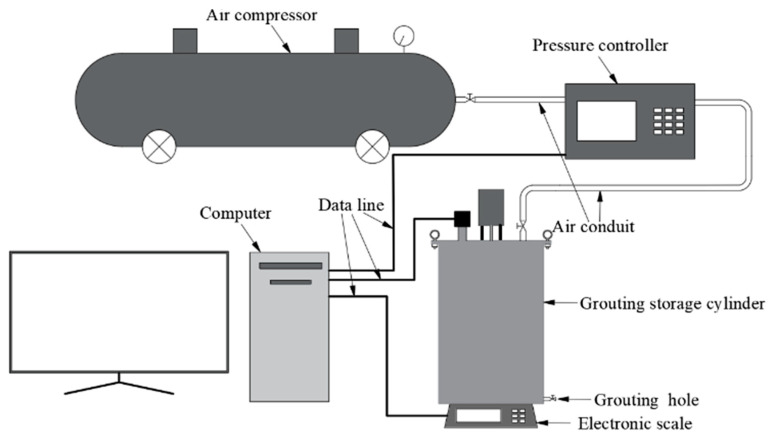
Schematic diagram of grouting equipment.

**Figure 6:**
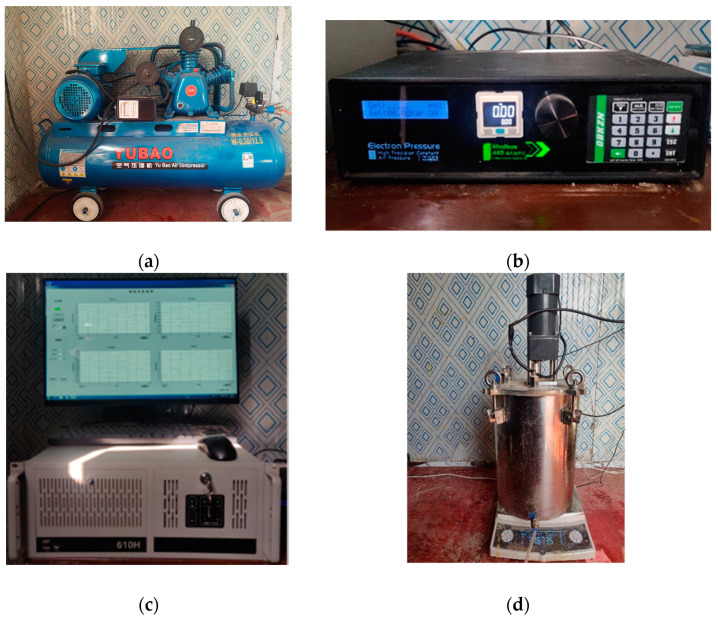
Photograph of grouting equipment: (**a**) air compressor; (**b**) intelligent pressure controller; (**c**) control computer; and (**d**) hopper.

The grouting equipment adopts an independently designed constant pressure intelligent two-liquid grouting device; the equipment mainly consists of an air compressor, intelligent digital constant pressure controller, control computer, storage barrel, and other parts; the advantage of this equipment is that the control computer of this equipment can perform real-time control of the internal pressure of the storage barrel to realize the constant pressure grouting; the schematic diagram of this equipment is shown in Figure 5. Figure 6 shows the photograph of grouting equipment.

**Figure 7:**
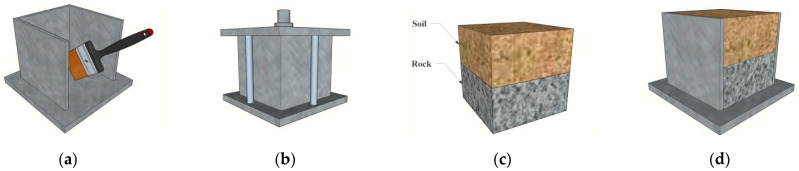
Photos of production process for specimens: (**a**) apply mold-release oil; (**b**) grouting test mold; (**c**) soil and stone contact; and (**d**) place in mold.

Figure 7 shows the specimen molding process diagram and the first test of soil air-drying through the 2 mm sieve. First, the moisture content of the amount of water required to add is calculated, weighed after the water is uniformly sprayed onto the soil samples and mixed, and then will be blended with a good moisture content of the soil into the sealing bag and placed in an airtight container for 24 h so that the water is fully infiltrated. After the soil samples are prepared, the subsequent specific grouting reinforcement test steps are as follows:

1. Apply a layer of mold-release oil on the inner wall of the grouting mold, affix a layer of tinfoil to facilitate the late demolding, and ensure that there are no air bubbles and wrinkles in the process of tinfoil affixing so as to prevent the influence on the outer shape of the test piece;
2. The test specifications of the concrete specimen are placed on the bottom of the mold, to ensure that the concrete cutting surface faces upward and makes soil contact, and then according to the test set, the dry density of the measured test soil is poured into the mold; using the layered compaction method, each layer of the specimen height should be equal to the junction of the soil layer and should be shaved to prevent the soil stratification;
3. Tighten the mold’s fixed screw, open the grouting valve above the mold, and then connect to the constant pressure grouting equipment; grouting is completed after closing the grouting valve above the mold. In this test, pressure grouting was used to bond the polymer to the soil-rock interface.
4. Grouting the completed mold is performed at a constant temperature of 20 °C for the solidification reaction and hardened 3 h after the slurry; remove the model to take out the molding specimen and mark and place it in a constant temperature room for natural maintenance for 7 days.

## 3. Results

### 3.1. Interface Shear Strength and Shear Displacement Curves

**Figure 8:**
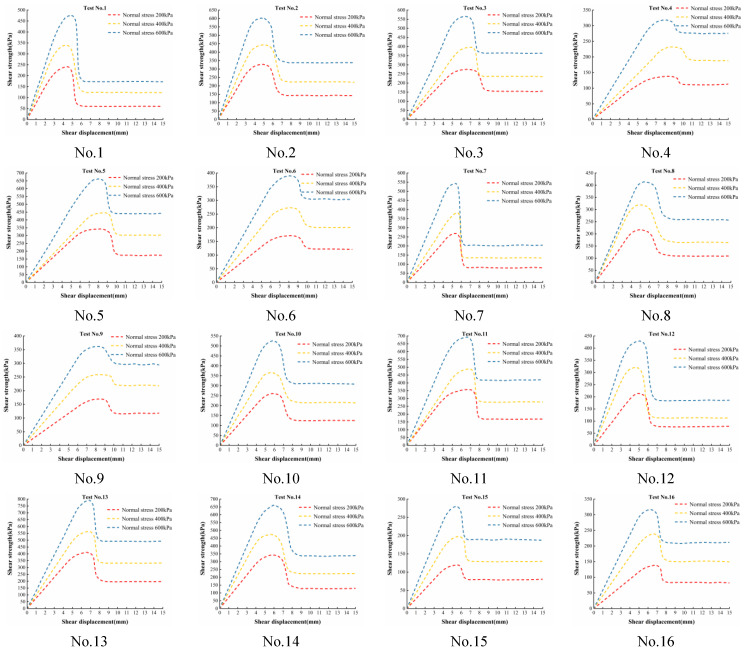
Interface shear strength and shear displacement curves.

The permeable polymer can effectively fill the pores of soil particles and penetrate into the concrete-soil interface, cement the soil particles together through a chemical reaction with water in the soil, and form chemical adhesion at the interface [34,35]. Figure 8 shows the interfacial shear strength and shear displacement curves for each of the 16 test numbers in the orthogonal design table under three normal stresses. In this paper, the normal stress is set according to the actual situation, with the aim of investigating the effect of bond properties at the concrete-soil interface after polymer grout repair.

**Figure 9:**
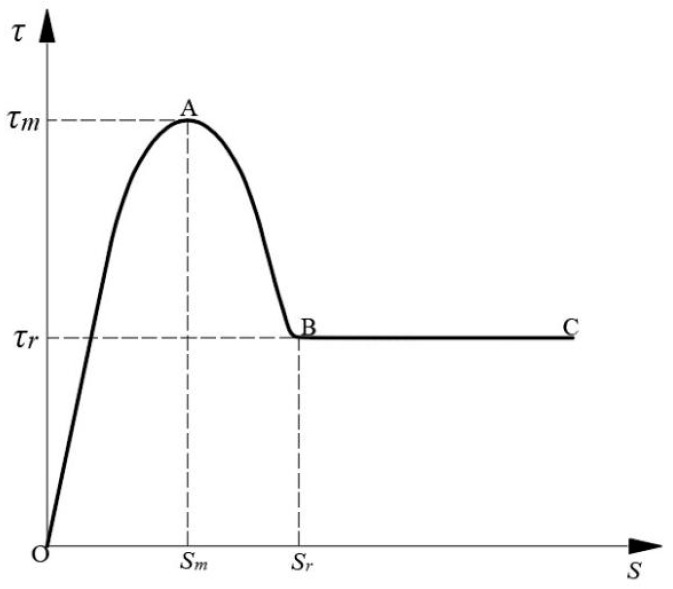
Interfacial shear strength-tangential displacement curve diagram.

The interfacial shear strength increases with the increase in normal stress, while the initial tangent modulus and the peak cut-off modulus of the curves both increase with the increase in normal stress. The shear strength-shear displacement curve of the interface has obvious geometrical characteristics, i.e., presenting the shear strength firstly increases with the increase in shear displacement, and when the peak shear strength is reached, the interfacial shear strength decreases with the increase in shear displacement and finally reaches the nearly horizontal residual shear strength. Therefore, in this paper, the interfacial shear strength-shear displacement curve under normal stress is simply divided into three stages, i.e., the rising section, the falling section, and the residual section, presenting the trend shown in Figure 9.

In Figure 9, point A is the peak shear strength coordinate point, i.e., the vertical coordinate is the peak shear strength *τ_m_*, and the horizontal coordinate is the shear displacement corresponding to the peak shear strength *S_m_*; point B is the initial coordinate point of the residual shear strength stage, i.e., the vertical coordinate is the residual shear strength *τ_r_*, and the horizontal coordinate is the limiting shear displacement at the interface *S_r_*. Therefore, the characteristic parameters of the interface shear strength-shear displacement curves are mainly the shear strengths *τ_m_* and *τ_r_*, and shear displacements *S_m_* and *S_r_*.

The shear fracture energy is used to characterize the energy consumed in the fracture process [36,37,38]. The interfacial shear fracture energy represents the energy required to fracture the material and is calculated as

(2) $$W=\int_{0}^{S_{r}}\tau \left(s\right)ds$$

where *W* is the shear fracture energy of the interface, *τ*(*s*) is the interface shear strength-shear displacement curve. The above formula mainly calculates the area enclosed by the shear strength-shear displacement curve and the horizontal coordinate axis in the shear fracture displacement range (0~*S_r_*) of the interface, which is the shear fracture energy of the interface.

Table 4 shows the characteristic parameters of each test number for orthogonal tests under three normal stresses *S_r_* and *S_m_*.

The results of the interfacial shear fracture energy calculated using Equation (2) are shown in Table 5. From Table 5, the interfacial shear fracture energy increases to different degrees with the increase in normal stress.

**Figure 10:**
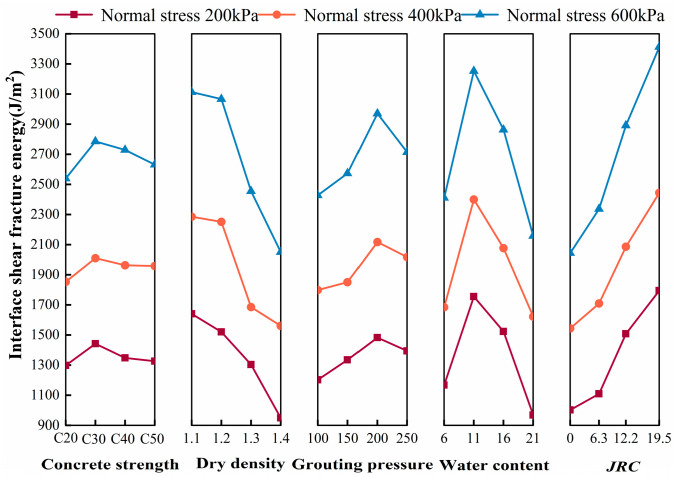
Trends in the influence of factors on interfacial shear fracture energy.

From Figure 10, with the increase in interfacial roughness (*JRC*) and grouting pressure, the interfacial shear fracture energy increases, which is mainly due to the fact that the interfacial peak shear strength and residual shear strength increase with the increase in interfacial roughness and grouting pressure; in addition, with the increase in water content, the interfacial shear fracture energy also shows the tendency of increasing and then decreasing; with the increase in dry density, the interfacial shear fracture energy decreases; finally, the influence of concrete strength on interfacial shear fracture energy is still not obvious, which is mainly due to the fact that the interface and cured soil shear damage surface is basically lower than the dry density. With the increase in dry density, the interfacial shear fracture energy decreases, mainly due to the lower interfacial shear strength as the dry density increases; finally, the influence of the concrete strength on the interfacial shear fracture energy is still insignificant, which is mainly due to the fact that the interfacial shear damage is basically at the interface and the internal cured soil; although a small amount of damage occurs on the surface of the concrete, the damaged area is very small.

**Figure 11:**
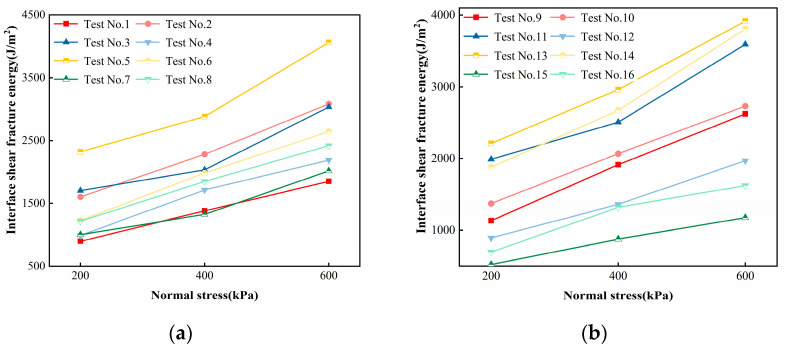
Relationship between normal stress and interfacial shear fracture energy: (**a**) Test 1 to Test 8 and (**b**) Test 9 to Test 16.

Figure 11 shows the relationship between normal stress and interfacial shear fracture energy. Normal stress and interfacial shear fracture energy are approximately linear, and orthogonal Test No. 5 has the largest sum of shear fracture energies at three normal stresses and orthogonal Test No. 15 has the smallest sum of shear fracture energies at three normal stresses. The sum of the shear fracture energies under the three normal stresses was the largest for orthogonal Test No. 5 and the smallest for orthogonal Test No. 15, with a difference of 3.6 times.

### 3.2. Shear Failure Model

**Figure 12:**
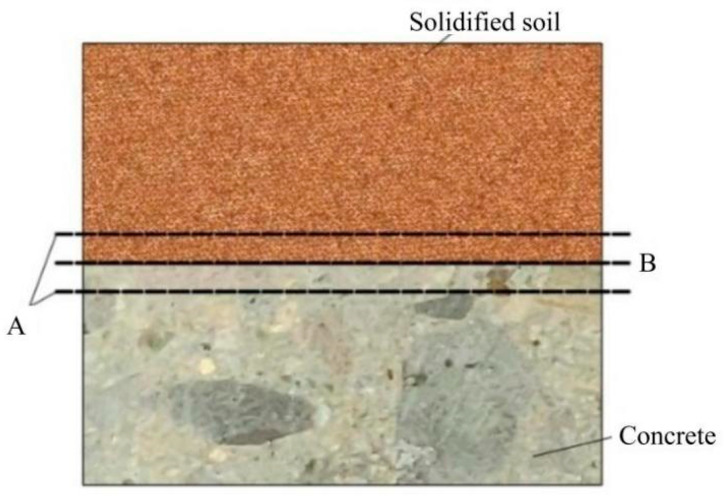
Schematic diagram of the interface destruction model.

**Figure 13:**
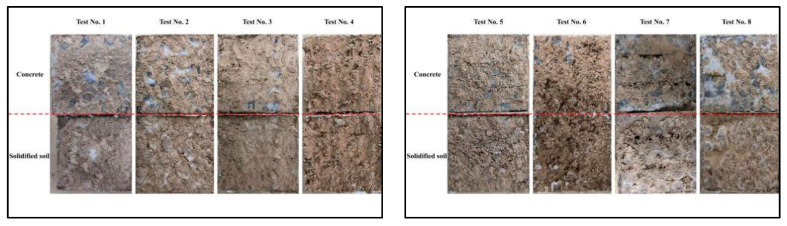

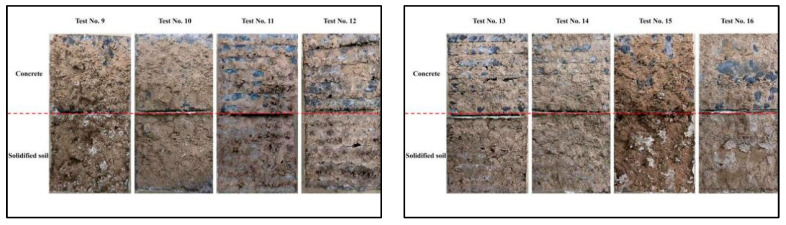
Interface after shear damage.

Figure 12 shows the Schematic diagram of the interface destruction model. The location where interfacial shear damage occurs exists in two cases, the first is interfacial damage between the base materials and the second is damage that occurs at the base materials; when the interface bond strength is greater than the matrix material strength, the damage surface mainly occurs inside the matrix; otherwise, the damage surface occurs at the interface [39]. According to the related studies [18,24] and test results, the defined concrete-soil interface damage modes after grouting are mainly divided into two types, which are the damage occurring inside the matrix material (type A) and the interface damage (type B). Figure 13 shows the interface after shear damage. The concrete surface after shear damage is adhered with cured soil, and the shear damage modes are all composite types of A-type and B-type damage, and the A-type damage almost always occurs inside the cured soil, which is due to the fact that the intrinsic shear strength of the concrete substrate is greater than that of the interface and the interior of the cured soil substrate material, and therefore, the damage area occurring inside the concrete substrate material is very small in the actual test process, so the A-type damages in this paper are all considered to have occurred inside the cured soil.

**Figure 14:**
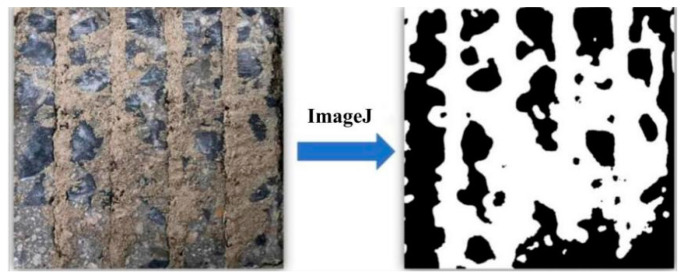
ImageJ interface processing example.

In order to quantitatively analyze the interface damage pattern, ImageJ image processing software V1.6.4 was used to analyze and calculate the area of adherent soil on the concrete surface after interface shear damage [40,41,42,43]. Therefore, ImageJ software was used to binarize the concrete surface after shear damage and then calculate the area of adherent soil on the surface. The processing of ImageJ software is shown in Figure 14, where the white area is the adherent soil on the surface of the concrete, and the black area is the concrete, and the results of the calculation are shown in Table 6.

From Table 6, there is a large area of adherent soil on the concrete surface after shear damage, and the percentage of adherent soil area is above 40%, and the area of adherent soil at the interface increases with the increase in normal stress. The data in Table 6 show that the permeable polymer used in this paper has strong chemical adhesion, and in the process of shear damage, the damage surface mostly occurs in the interior of the cured soil, which is due to the permeable polymer penetrating into the connecting holes and through holes in the soil under the action of grouting pressure, but it cannot penetrate into the blind holes and closed holes in the soil, and at the same time, due to the bond strength of the interface being larger. At the same time, due to the modulus mismatch and irregular geometry, the internal stress concentration phenomenon will occur in the cured soil during the shear process, resulting in internal damage to the cured soil.

**Figure 15:**
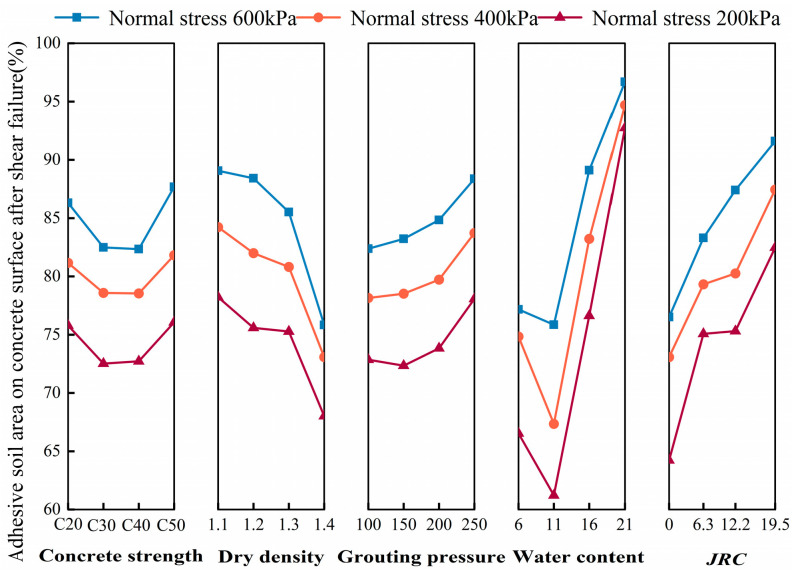
Trends in the influence of factors on interfacial damage patterns.

Figure 15 presents a graphical representation of the extreme difference analysis of the interface damage mode. The influence of water content on the trend is primarily attributed to the utilization of grouting material properties. The water content exerts a direct impact on the chemical adhesion of the interface. The reduction in chemical adhesion results in a corresponding reduction in the percentage of the area of the interface adhering to the soil after shear damage. The shear damage mode undergoes a gradual transition from type A to type B. Furthermore, this paper demonstrates that the concrete surface groove can be used to control the interface roughness. The slurry, under the influence of grouting pressure, infiltrates into the concrete surface groove, curing soil particles simultaneously. This process results in the bonding of soil particles with the concrete surface, thereby increasing the bond strength of the interface. Consequently, the ratio of the area of shear damage to the adherent soil increases, and the interface shear damage mode transitions gradually from type B to type A. The dry density and grouting pressure influence the penetration effect of the slurry at the interface. A greater dry density results in a more pronounced reduction in penetration effect, which in turn leads to a weaker interfacial bond strength. Consequently, the interfacial shear damage mode gradually transitions from type A to type B. It can be observed that as the grouting pressure increases, the penetration effect improves, and the interfacial bond strength increases. Consequently, the interfacial shear damage mode transitions from type B to type A. Furthermore, since the internal strength of the concrete is greater than the interfacial strength and the strength of cured soil, the relationship between the shear damage mode and the strength of the concrete is not evident. The order of the factors affecting the percentage of the adhered soil area on the concrete surface after shear damage under three normal stresses is as follows: water content > interface roughness > dry density > grouting pressure > concrete strength.

**Figure 16:**
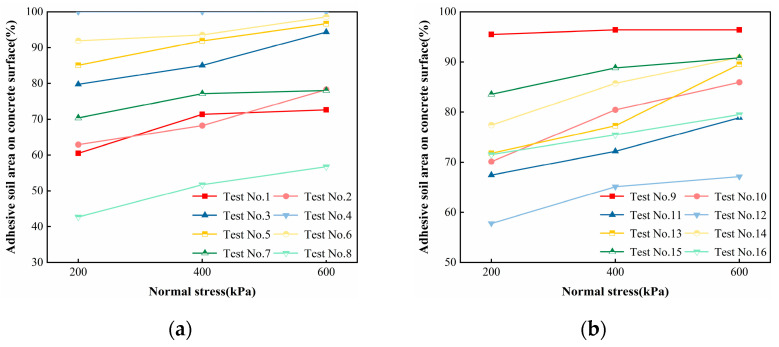
Relationship between normal stress and the percentage of adherent soil area on the concrete surface after shear damage: (**a**) Test 1 to Test 8 and (**b**) Test 9 to Test 16.

Figure 16 shows the relationship between the area percentage of adherent soil on the concrete surface after shear damage and normal stress, from which it can be seen that with the increase in normal stress, the area percentage of adherent soil on the concrete surface after shear damage basically becomes an increasing trend. This is mainly due to the infiltration grouting process; the slurry cannot penetrate into the blind holes and closed holes in the soil body; there is insufficient infiltration of the cured soil inside the region, and at the same time, in the normal stress loading, the cured soil itself produces compressive deformation, resulting in the internal structure of the cured soil in the shear before there is damage in the cured soil internal structure; in the shear loading of the internal structure, the cured soil is prone to produce a concentration of stress and lead to further expansion of the damage, and therefore, the interface damage. Therefore, the interface damage mode transitions from type B to type A. The relationship between normal stress and the percentage of adhered soil area on the concrete surface following shear damage is not particularly sensitive. This is primarily due to the fact that normal stress does not represent a significant factor influencing the percentage of the adhered soil area on the concrete surface following shear damage.

### 3.3. Study of the Whole Process of Interfacial Shear Damage

#### 3.3.1. Displacement Analysis of the Whole Process of Interfacial Shear Damage

**Figure 17:**
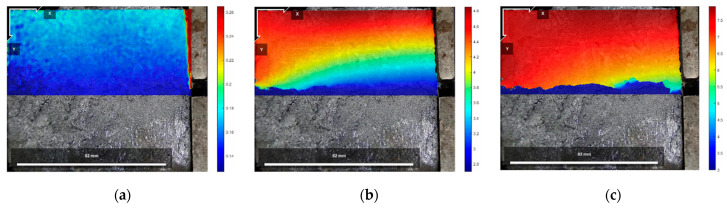
Horizontal displacement cloud at 200 kPa normal stress: (**a**) initial state before shear; (**b**) peak shear strength phase; and (**c**) residual shear strength state.

**Figure 18:**
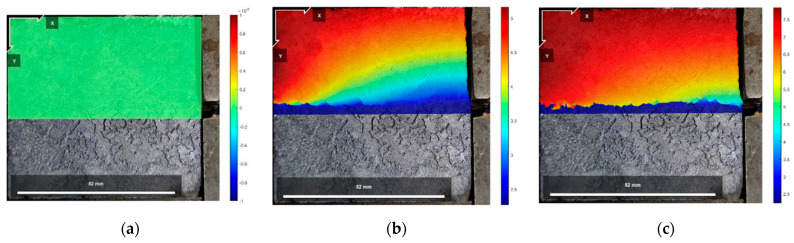
Horizontal displacement cloud at 400 kPa normal stress: (**a**) initial state before shear; (**b**) peak shear strength phase; and (**c**) residual shear strength state.

**Figure 19:**
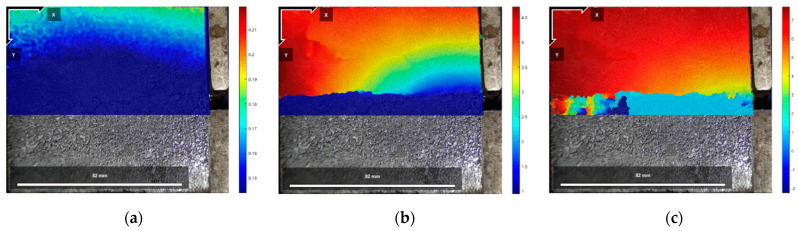
Horizontal displacement cloud at 600 kPa normal stress: (**a**) initial state before shear; (**b**) peak shear strength phase; and (**c**) residual shear strength state.

The DIC digital image correlation technique was effectively used to analyze the material deformation and damage process [44,45,46,47]. Since Test No. 2 was more effective and representative of the general situation, the initial state before shear, the peak shear strength stage, and the residual shear strength stage of the test specimen of Test No. 2 during the direct shear test under different normal stresses were selected for analysis, of which Figure 17, Figure 18 and Figure 19 are the horizontal displacement cloud diagrams under the three normal stresses, respectively.

Figure 17a, Figure 18a and Figure 19a show the initial state before shear. From Figure 17b, Figure 18b and Figure 19b, the cured soil near the loading end at the same horizontal position during the peak shear strength stage has a larger horizontal displacement relative to the far end, and a localized shear damage surface has appeared near the loading end, while the interface at the far end from the loading end is still in the bonded state. Therefore, with the loading of the shear load, the cured soil near the loading end is compressed to produce horizontal displacement; the horizontal displacement is gradually transferred from the loading end to the right; the interface near the loading end is the first to produce damage, but the interface has not yet formed a penetrating damage surface in the stage of the peak shear strength, and with the further loading, the shear damage surface is rapidly expanding, and in the stage of the residual shear strength, the interface has been the complete shear damage surface; the interface has been the bond failure, and the cured soil interface is still in the bond state. The bond failure and the internal displacement of the cured soil are large. Figure 17c, Figure 18c and Figure 19c show the residual shear strength state.

**Figure 20:**
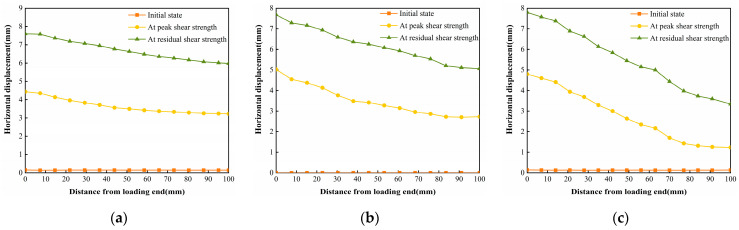
Displacements in the horizontal direction for different states of the interface under three normal stresses: (**a**) normal stress 200 kPa; (**b**) normal stress 400 kPa; and (**c**) normal stress 600 kPa.

The horizontal displacement data of three states in Figure 17, Figure 18 and Figure 19 can be extracted, and the horizontal displacement map of cured soil at the 0.5 cm horizontal position above the shear damage surface can be derived, and it can be seen from Figure 20 that the closer the distance from the loading end, the larger the horizontal displacement, and with the increase in the distance from the loading end, the horizontal displacement gradually decreases. Table 7 shows the horizontal displacement data in different states of interface shear under three normal stresses, from which it can be seen that, with the increase in normal stress, the ratio of the horizontal displacement of the cured soil at a distance of 100 mm from the loading end to that at 0 mm is gradually becoming smaller, and the ratio is larger than that of the peak shear strength at the stage of residual shear strength under the same normal stress.

The reason is that in the peak shear strength stage, the interface near the loading end produces a localized shear damage surface, and the distal end of the loading end still has part of the interface in a bonded state, so the horizontal displacement of the distal end of the cured soil affected by the effect of interfacial bonding is relatively small, but in the residual shear strength stage, the interface has been penetrating the damaged surface, and the interface is completely bonded to the failure, so the ratio of the peak shear strength stage is smaller than the peak shear strength stage. In the residual shear strength stage, the ratio is smaller than that in the peak shear strength stage.

**Figure 21:**
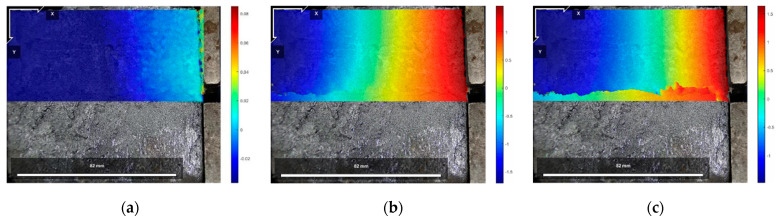
Vertical displacement cloud at 200 kPa normal stress: (**a**) initial state before shear; (**b**) peak shear strength phase; and (**c**) residual shear strength state.

**Figure 22:**
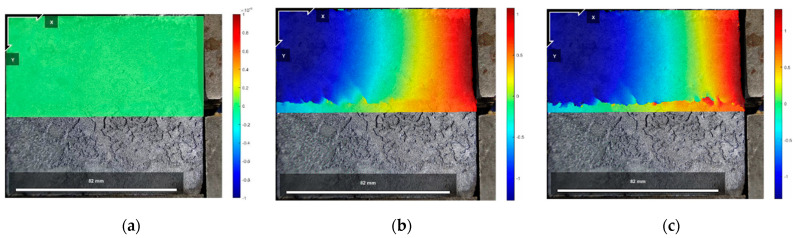
Vertical displacement cloud at 400 kPa normal stress: (**a**) initial state before shear; (**b**) peak shear strength phase; and (**c**) residual shear strength state.

**Figure 23:**
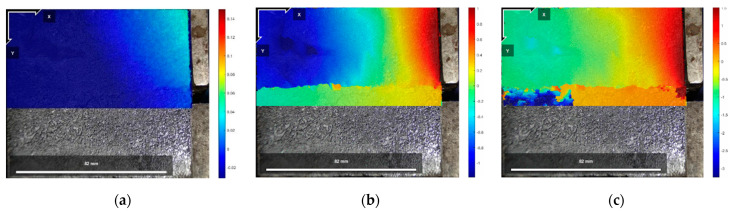
Vertical displacement cloud at 600 kPa normal stress: (**a**) initial state before shear; (**b**) peak shear strength phase; and (**c**) residual shear strength state.

Figure 21, Figure 22 and Figure 23 show the vertical displacement cloud diagrams of the Test No. 2 specimen under three normal stresses in the initial state before shear: the peak shear strength stage and the residual shear strength stage, from which it can be seen that the cured soil at the proximal end of the loading end bulges upward under the shear load, and at the distal end of the loading end, the cured soil is compressed, and the cured soil exists in the middle of the position of the cured soil in the area of the vertical displacement is zero. And, it can be seen from Figure 21b, Figure 22b and Figure 23b that at the peak shear strength stage, the interface of the uplifted region of the cured soil shows a more obvious shear damage surface, while the interface of the compressed region of the cured soil is still in the bonded state.

**Figure 24:**
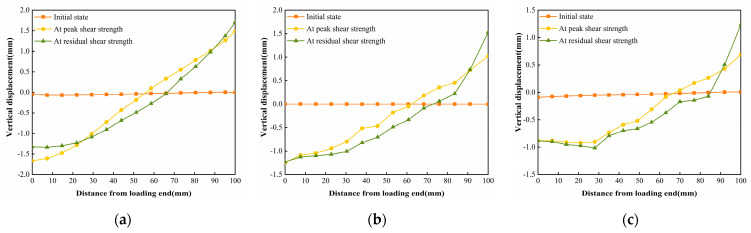
Displacements in the vertical direction for different states of the interface under three normal stresses: (**a**) normal stress 200 kPa; (**b**) normal stress 400 kPa; and (**c**) normal stress 600 kPa.

The vertical displacement data in Figure 21, Figure 22 and Figure 23 were extracted from the three states, and the vertical displacement of the cured soil at the horizontal position of 0.5 cm above the shear surface could be derived, and it can be seen from Figure 24 that, with the increase in the distance from the loading end, the vertical displacement of the cured soil was gradually changed from bulging to downward compression, and the distance from the loading end corresponding to the vertical displacement at zero in the stage of residual shear strength was larger than that in the stage of peak shear strength; with an increase in the normal stress, the maximum vertical bulging displacement and the maximum vertical displacement in the process of shear increased. In addition, the vertical uplift and compression displacements of the cured soil in the vertical direction under the three normal stresses in Figure 24 do not exceed 2 mm.

The reason for the above phenomenon is that in the peak shear strength stage, the cured soil near the loading end is compressed horizontally under the shear load, and the cured soil rises upward, while the cured soil at the far end of the loading end has a smaller horizontal displacement and is compressed vertically by the influence of the normal stress and the interface bonding, and the interface appears to have a penetrating damage surface in the stage of residual shear strength, and the interface fails to be bonded completely and becomes frictional, so the vertical deformation at zero at the stage of residual shear strength is greater than that at the stage of peak shear strength. Therefore, the distance from the loading end when the vertical deformation is zero in the residual shear strength stage is larger than that in the peak shear strength stage; at the same time, with the increase in normal stress, the increase in interfacial friction will impede the increase in the horizontal displacement of the cured soil at the distal end of the loading end, so the horizontal compression of the cured soil at the proximal end of the loading end is increased, and the distance from the loading end corresponding to the time when the vertical displacement is zero is increased as well.

#### 3.3.2. Strain Analysis of the Whole Process of Interfacial Shear Damage

**Figure 25:**
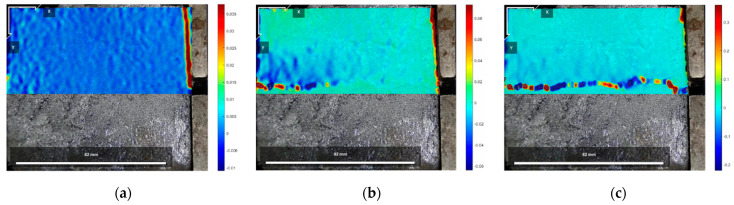
Exx strain cloud at 200 kPa normal stress: (**a**) initial state before shear; (**b**) peak shear strength phase; and (**c**) residual shear strength state.

**Figure 26:**
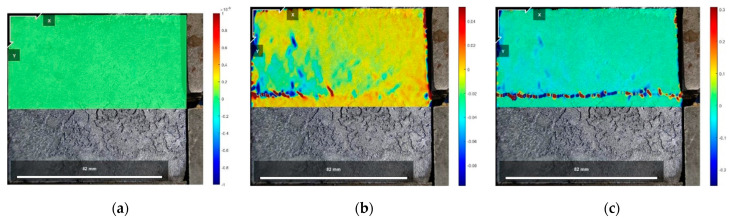
Exx strain cloud at 400 kPa normal stress: (**a**) initial state before shear; (**b**) peak shear strength phase; and (**c**) residual shear strength state.

**Figure 27:**
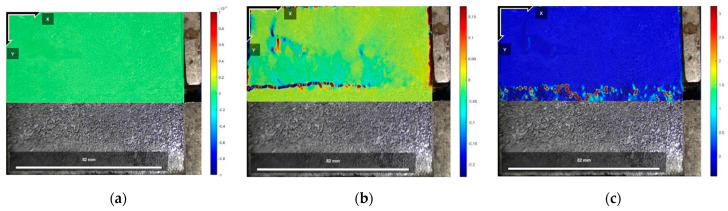
Exx strain cloud at 600 kPa normal stress: (**a**) initial state before shear; (**b**) peak shear strength phase; and (**c**) residual shear strength state.

Figure 25, Figure 26 and Figure 27 show the cloud diagrams of horizontal strain Exx in the initial state before shear, peak shear strength stage, and residual shear strength stage of the specimen of Test No. 2 under three normal stresses, respectively, from which it can be seen that horizontal compressive strains appeared in the cured soil above the damaged surface under the three normal stresses, and the further the horizontal compressive strains from the loaded end, the smaller the horizontal compressive strains are.

**Figure 28:**
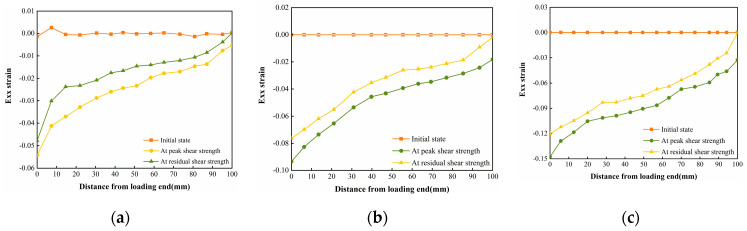
Horizontal strain Exx for different states of the interface under three normal stresses: (**a**) normal stress 200 kPa; (**b**) normal stress 400 kPa; and (**c**) normal stress 600 kPa.

The horizontal strain data in Figure 25, Figure 26 and Figure 27 were extracted from the three states, and the horizontal strain map of the cured soil at the horizontal position of 0.5 cm above the shear damage surface can be derived. From Figure 28, it can be seen that the horizontal compressive strain of the cured soil at the proximal end of the loading end increases with the increase in normal stress, which is mainly due to the fact that the vertical bulge displacement of the cured soil is at the proximal end of the loading end. This is mainly due to the fact that the vertical uplift displacement of the cured soil near the loading end decreases with the increase in normal stress; at the same time, the horizontal compressive strain in the peak shear strength stage is always larger than that in the residual shear strength stage, and the horizontal compressive strain in the residual shear strength stage is close to the initial state before shear at the furthest end from the loading end, while there is still a small horizontal compressive strain in the peak shear strength stage; this is because the interface has been completely damaged by the slippage during the residual shear strength stage, and the cured soil has produced stress release in the position of 100 mm away from the loading end. This is because at the residual shear strength stage, and the interface has completely slipped and broken down, and the cured soil at the location of 100 mm from the loading end produces stress relief without the influence of interfacial friction, while the cured soil at the distal end of the peak shear strength stage is still in the bond state.

**Figure 29:**
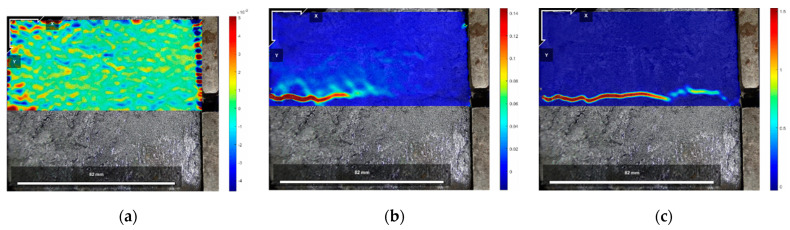
Eyy strain cloud at 200 kPa normal stress: (**a**) initial state before shear; (**b**) peak shear strength phase; and (**c**) residual shear strength state.

**Figure 30:**
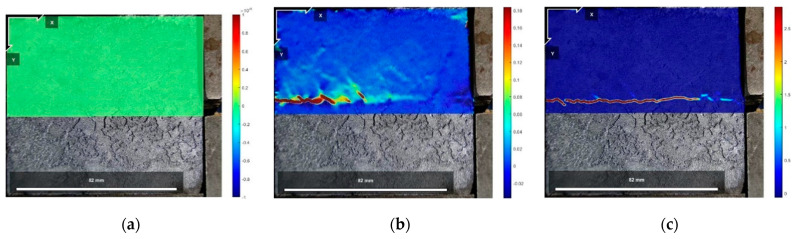
Eyy strain cloud at 400 kPa normal stress: (**a**) initial state before shear; (**b**) peak shear strength phase; and (**c**) residual shear strength state.

**Figure 31:**
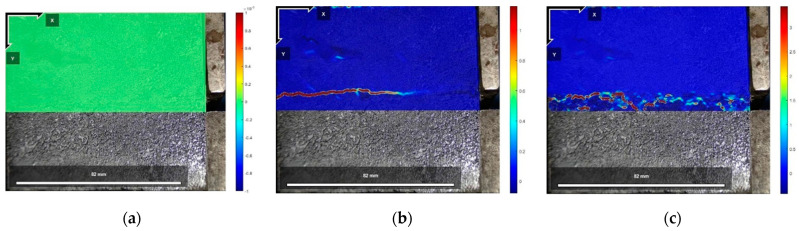
Eyy strain cloud at 600 kPa normal stress: (**a**) initial state before shear; (**b**) peak shear strength phase; and (**c**) residual shear strength state.

Figure 29, Figure 30 and Figure 31 show the vertical strain Eyy cloud plots of the specimen of Test No. 2 in the initial state before shear, peak shear strength stage, and residual shear strength stage under three normal stresses, respectively, from which it can be seen that, in the peak shear strength stage, vertical tensile strains appeared above the interfacial cracks near the loading end, and the further away from the cracks, the smaller the vertical tensile strains were, whereas, in the residual shear strength stage, the vertical tensile strains of the cured soil above the shear damage surface were smaller due to the stress release generated. The cured soil above the damaged surface has a smaller vertical tensile strain due to the stress release generated during the residual shear strength stage.

**Figure 32:**
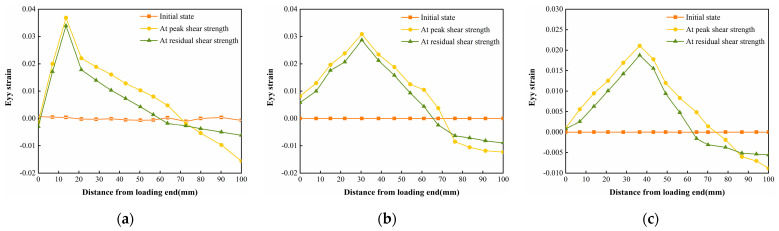
Vertical strain Eyy for different states of the interface under three normal stresses: (**a**) normal stress 200 kPa; (**b**) normal stress 400 kPa; and (**c**) normal stress 600 kPa.

The vertical strain data in the three states in Figure 29, Figure 30 and Figure 31 were extracted, and the vertical strain map of the cured soil at the horizontal position of 0.5 cm above the shear surface can be obtained, and it can be seen from Figure 32 that the vertical strains in the peak shear strength stage and the residual shear strength stage show a tendency of increasing first and then decreasing with the distance from the loading end, i.e., the vertical tensile strain is the vertical strain first and then the vertical compressive strain.

### 3.4. Analysis of Microtest Results

**Figure 33:**
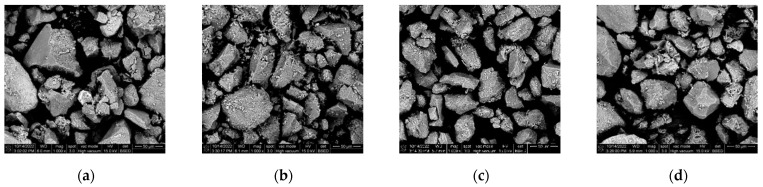
SEM images of the pulverized soil specimen before grouting: (**a**) Test No. 3; (**b**) Test No. 4; (**c**) Test No. 7; and (**d**) Test No. 13.

**Figure 34:**
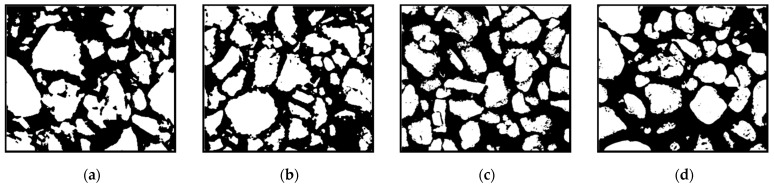
SEM images of ImageJ processed chalk specimen before grouting: (**a**) Test No. 3; (**b**) Test No. 4; (**c**) Test No. 7; and (**d**) Test No. 13.

Figure 33 shows the 1000× SEM images of the pulverized soil before grouting. Most of the pulverized soil particles before grouting existed in a single-grain state, with large pores. The microscopic images before grouting were binarized using ImageJ software. In Figure 34, the white area is the pulverized soil particles, and the black area is the pores between the soil particles. The apparent porosity was calculated, referring to the porosity calculated after the binarization of the SEM images. Figure 34 shows that the pore space between the soil particles before grouting was large, and the distribution of soil particles was more dispersed. The software calculations show that the apparent porosity of Test No. 3, Test No. 4, Test No. 7, and Test No. 13 before grouting were 48.66%, 49.39%, 51.30%, and 52.20%, respectively.

**Figure 35:**
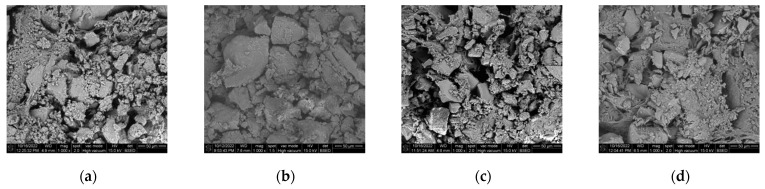
SEM images of cured soil after grouting: (**a**) Test No. 3; (**b**) Test No. 4; (**c**) Test No. 7; (**d**) and Test No. 13.

**Figure 36:**
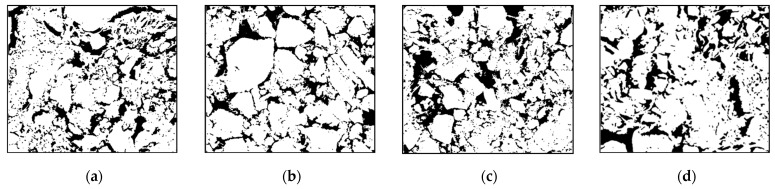
ImageJ processed SEM images of the consolidated soil after grouting: (**a**) Test No. 3; (**b**) Test No. 4; (**c**) Test No. 7; and (**d**) Test No. 13.

Figure 35 shows the microscopic picture of Figure 33 after grouting. Compared with the microstructure before grouting, the soil particles were effectively filled by the slurry after grouting, and the pore size decreased significantly. Thus, the larger soil particles were bonded together by the slurry to form larger agglomerates, and smaller soil particles were wrapped up by the slurry. Figure 36 shows the images after ImageJ processing. Compared with the pulverized soil particles before grouting in Figure 34, the pulverized soil particles after grouting were bonded together by the slurry from Figure 36 and became agglomerates from the original single-grain state. The pore size was obviously smaller. Using ImageJ software, the apparent porosity of Test No. 3, Test No. 4, Test No. 7, and Test No. 13 after grouting in Figure 36 were 22.64%, 20.35%, 24.26%, and 23.95%, respectively. Therefore, the permeable polymer can significantly reduce the porosity of the pulverized soil.

**Figure 37:**
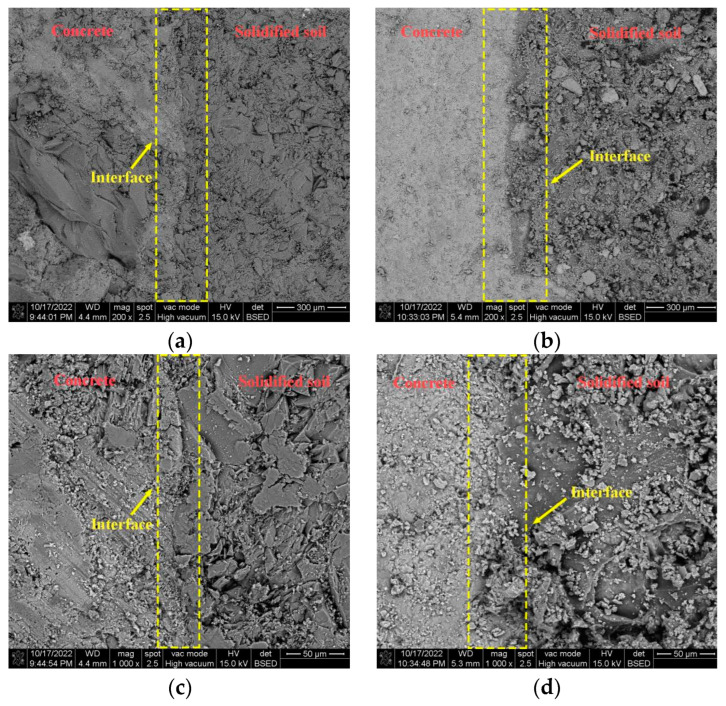
ImageJ processed SEM images of the consolidated soil after grouting: (**a**) magnification 200× image after grouting; (**b**) magnification 200× image after grouting; (**c**) magnification 1000× image after grouting; and (**d**) magnification 1000× image after grouting.

Figure 37 shows the SEM images of the interface after grouting in Test No. 7 and Test No. 13, respectively, from which it can be seen that the permeable polymers can effectively fill the soil particles and tightly bond the two materials, soil and concrete, together [34]. It can also be seen that there is a layer of cementing layer with a small thickness mainly composed of permeable polymer on the right side of the interface, so it can be seen that the slurry penetrates into the concrete surface through the pores of soil particles under the action of the grouting pressure to form a layer of cementing layer of a very small thickness on the surface of the concrete, which mainly consists of the slurry, and it is tightly bonded with the surface of the concrete to form the interface bonding.

According to the above analysis, the polymer is able to reinforce the concrete-soil interface by penetrating into the soil and reaching between the concrete-soil interface to form a bond with a certain strength, and the bonding mode of the concrete-soil after grouting is the concrete-cohesive layer-cured-soil mode.

## 4. Conclusions

In this paper, based on the penetration grouting technology to reinforce the interface between concrete and the surrounding soil, the orthogonal test was used to explore the influence law of different factors on the bonding characteristics of the concrete-soil interface after grouting through the direct shear test, and at the same time, the whole process of the evolution of the shear damage of the concrete-soil interface after grouting was analyzed using the DIC technology in the course of the direct shear test; then, the microstructure of the solidified soil and bonded interface was analyzed through the SEM test for the pre-grouting and post-grouting periods. The microstructures of the cured soil and bonded interface were analyzed by the SEM test, and the intrinsic mechanism of grouting reinforcement and the micro-mechanism of water damage in the submerged environment were explored, and the main conclusions are as follows:

1. By applying grouting pressure, the permeable polymer effectively fills the soil particle pores and infiltrates the interface between the soil and rock. The chemical reaction leads to the creation of interfacial cohesion.
2. The interfacial shear fracture energy is calculated from the interfacial shear strength-shear displacement curve, and the factors affecting the interfacial shear fracture energy are in the following order of priority: interfacial roughness > water content > dry density > grouting pressure > concrete strength, and at the same time, with the increase in the normal stress, the interfacial shear fracture energy is also increased.
3. We quantitatively analyzed the change in interface damage pattern by ImageJ, and concluded that the factors affecting the interface damage pattern are in the following order: water content > interface roughness > dry density > grouting pressure > concrete strength, and with the increase in normal stress, the percentage of the area of adherent soil on the surface of the concrete after shear damage basically shows a tendency to increase.
4. Using the DIC technique in the process of the direct shear test, it can be seen through the analysis of the displacement and strain data during the whole process of interface shear damage that the horizontal displacement and horizontal compressive strain of the cured soil at the distal end of the loading end decreases gradually with the increase in normal stress in the process of shear, and the maximum vertical uplift displacement and maximum vertical tensile strain of the cured soil at the proximal end of the loading end.
5. Some test numbers in the orthogonal test were selected for microtests to investigate the micro-mechanism of the concrete-soil interface after grouting. From the SEM images, it can be seen that the polymer penetrates into the concrete-soil interface and forms a cementing layer, which makes the concrete-soil interface have a strong bond, and the bonding form is in the mode of concrete-cohesive layer-cured soil.

## Figures and Tables

**Table 1:** Physical properties of soil.

Property	Value	The Standards Used
Specific gravity G_S_	2.70	Geotechnical engineering test method and criterion (GB/T50123-2019) [5]
Percent moisture content of liquid limit *w*_L_/%	24.00	
Percent moisture content of plastic limit *w*_P_/%	16.90	
Plasticity index I_P_	7.10	
Natural moisture content/%	11.80–19.37	
Maximum dry density ρdmax/g·cm^−3^	1.44	

**Table 2:** Properties and composition of permeable polyurethane.

Viscosity 23 ± 2 °C (mPa·s)	Density (g/cm^3^)	Compressive Strength (MPa)	Components	Viscosity 23 ± 2 °C (mPa·s)	Density (g/cm^3^)
10	1.1	2.5	A	4	1.05
			B	23	1.20

**Table 3:** Interface roughness table.

Roughness of Surface	Surface Grooving Method	*JCR*
Smoothness	Not dealt with	0
Roughness 1	Depth 3 mm, width 2 mm, length 100 mm, slot spacing 20 mm	6.3
Roughness 2	Depth 6 mm, width 2 mm, length 100 mm, slot spacing 10 mm	12.2
Roughness 3	Depth 6 mm, width 2 mm, length 100 mm, slot spacing 50 mm	19.5

**Table 4:** Characteristic parameters of orthogonal tests for each test number.

Test Number	Normal Stress 200 kPa		Normal Stress 400 kPa		Normal Stress 600 kPa	
	*S_m_* (mm)	*S_r_* (mm)	*S_m_* (mm)	*S_r_* (mm)	*S_m_* (mm)	*S_r_* (mm)
1	4.43	6.13	4.26	6.58	4.92	6.32
2	4.88	7.56	5.01	7.79	4.71	7.78
3	6.65	9.49	7.07	8.46	6.48	8.51
4	8.37	10.55	8.72	11.64	7.88	10.45
5	8.32	10.52	8.74	10.47	8.10	10.02
6	8.18	10.87	8.07	11.12	8.08	10.46
7	5.34	6.69	5.59	6.30	5.40	6.41
8	4.89	8.92	4.88	8.98	5.18	8.98
9	8.46	10.73	8.63	11.08	8.06	10.96
10	5.77	8.59	5.55	8.90	5.79	8.27
11	6.84	8.47	6.86	8.35	6.64	8.11
12	4.93	7.14	4.63	6.92	5.06	7.12
13	6.56	8.78	6.79	8.54	6.77	8.29
14	5.77	8.93	5.61	8.84	5.90	9.06
15	5.51	6.89	5.75	7.07	5.44	6.76
16	6.72	8.34	6.49	8.42	6.30	8.16

**Table 5:** Interface shear fracture energy.

Test Number	Interface Shear Fracture Energy (J/m^2^)		
	Normal Stress 200 kPa	Normal Stress 400 kPa	Normal Stress 600 kPa
1	896.39	1382.23	1850.05
2	1604.05	2281.08	3083.28
3	1701.91	2035.24	3034.06
4	989.97	1713.79	2189.13
5	2320.66	2883.01	4062.51
6	1226.8	1984.31	2644.26
7	1002.63	1323.93	2017.27
8	1217.01	1848.98	2418.85
9	1134.94	1913.85	2622.73
10	1373.34	2068.5	2730.92
11	1989.94	2506.85	3592.27
12	893.31	1361.68	1970.3
13	2210.15	2962.23	3916.35
14	1877.87	2672.69	3806.92
15	521.23	876.8	1176.72
16	695.52	1320.09	1624.57

**Table 6:** Percentage of area of soil adhering to the concrete surface after shear damage.

Test Number	Percentage of Area of Soil Adhering to the Concrete Surface After Shear Damage (%)		
	Normal Stress 200 kPa	Normal Stress 400 kPa	Normal Stress 600 kPa
1	60.51	71.35	72.61
2	62.9	68.23	78.28
3	79.74	85.05	94.36
4	100	100	100
5	85.08	91.88	96.67
6	91.91	93.57	98.56
7	70.36	77.17	78.04
8	42.73	51.72	56.72
9	95.49	96.39	97.41
10	70.13	80.44	85.92
11	67.43	72.19	78.88
12	57.81	65.13	67.13
13	71.79	77.28	89.54
14	77.41	85.72	90.9
15	83.56	88.82	90.8
16	71.53	75.48	79.48

**Table 7:** Horizontal displacements for different states under three normal stresses.

Distance from Loaded End (mm)	Normal Stress 200 kPa		Normal Stress 400 kPa		Normal Stress 600 kPa	
	Peak Shear Strength Phase	Residual Shear Strength Stage	Peak Shear Strength Phase	Residual Shear Strength Stage	Peak Shear Strength Phase	Residual Shear Strength Stage
100	3.23	5.98	2.73	5.06	1.24	3.34
0	4.44	7.6	5.02	7.68	4.8	7.8
Specific value	72.75%	78.68%	54.38%	65.89%	25.83%	42.82%

## Data Availability

The data presented in this study are available within this article.
